# Feeding styles of caregivers of children 6-23 months of age in Derashe special district, Southern Ethiopia

**DOI:** 10.1186/1471-2458-12-235

**Published:** 2012-03-23

**Authors:** Mekitie Wondafrash, Tseganeh Amsalu, Mirkuzie Woldie

**Affiliations:** 1 Department of Population and Family Health, Jimma University, Jimma, Ethiopia; 2 Department of Food Safety and Food Quality, Faculty of Bioscience Engineering, Ghent University, Ghent, Belgium; 3 MercyCorps Ethiopia, Arba Minch, Ethiopia; 4 Department of Health Services Management, Jimma University, Jimma, Ethiopia

## Abstract

**Background:**

Apart from basic determinants, appropriate child care practices are important in prevention of growth faltering and undernutrition. Providing safe and appropriate quality complementary foods is crucial to child growth and development. However, some children in low-income communities grow normally mainly due to proper caregiver feeding behaviors. Hence, the objective of this study was to determine caregivers' feeding styles as well as to indentify predictors in Derashe special district, Southern Ethiopia.

**Methods:**

A community based cross-sectional study design was employed in the seven randomly selected Kebeles (smallest administrative unit) of Derashe special district. A total of 826 caregivers provided data pertaining to socio-demographic variables. However, 764 caregivers had complete data for the outcome variable (caregiver feeding style). A multistage stratified sampling technique was used to identify study subjects. An adapted Caregiver's Feeding Styles Questionnaire (CFSQ) was used to gather information about caregivers' feeding styles. Multivariate multinomial logistic regression was employed to identify predictors of caregivers' feeding style.

**Results:**

The majority (80.6%) of caregivers were biological mothers. Nearly seventy-six percent of the caregivers practiced a responsive feeding style. Caregivers other than the biological mother favoured a laissez-faire feeding style, while caregivers residing in rural Kebeles were more responsive. Caregivers with a breastfeeding frequency of more than eight times predicted both laissez-faire (RRR = 1.88; 95% CI = 1.03-3.41) and controlling (RRR = 1.7; 95% CI = 1.02-2.85) feeding styles as compared to responsive feeding.

**Conclusion:**

Responsive feeding was the commonest style practiced by the caregivers. Many of the caregivers who were rural residents and birth parents have been responsive in child feeding. The instruments needed to be validated in the Ethiopian context and an additional prospective study based on direct observation of caregiver-child interactions is recommended.

## Background

Malnutrition is the largest risk factor in the world for disability and premature mortality among young children, especially in developing countries [1,2]. Although the condition is entirely preventable, malnutrition is a significant underlying factor in more than half of the deaths of young children in these countries [1,3]. Malnutrition is a background factor for deaths from diarrhea, measles, acute respiratory infection, meningitis and malaria [4]. In Ethiopia, 47%, 11% and 38% of children under five years of age are stunted, wasted and underweight, respectively [5]. An Ethiopian child is 30 times more likely to die by his or her fifth birthday than a child in Western Europe and the most common cause of child death is the interacting combination of malnutrition and infection [6].

Malnutrition is a complex phenomenon that stems from various underlying determinants, including a lack of optimal feeding practices for infants and young children. In UNICEF's conceptual framework for determinants of nutritional status, maternal and child care practices have been given due attention in addition to sufficient food supply at the household level, access to health services and a clean environment [7]. Some children in low-income countries with high rates of malnutrition grow normally due to better education and household management, or coping skills of their mothers [8]. Moreover, proper feeding practices, which ensure intake, are as important as the provision of complementary foods that meet nutritional requirements.

The caregiver-child interaction (feeding style) critically influences dietary intake on top of the dietary aspect of child feeding [9]. However, comparing feeding styles between studies prior to 1995 was difficult due to a lack of consistency in terminology and unclear definitions of feeding styles [10]. Later on, feeding styles were conceptualized and defined by Birch and Fisher as controlling, laissez-faire, and responsive feeding styles [11], and have been used in different researches [9,12,13].

The "controlling" feeding style occurs when a caregiver has complete control of when, what and how much the child eats, and includes restriction and force-feeding the child. In the "laissez-faire" feeding style the caregiver makes little effort to encourage eating; feeding is not encouraged even when the child may be marginally nourished [14]. Birch and Fisher defined the term "responsive" feeding as a condition in which "the caregiver is in close proximity to the young child during the meal and responds to the child's hunger cues in a reasonable time" [11]. Responsiveness is now viewed by different authors as a three-step process, namely observation of child cues by the caregiver, interpretation of the signs and taking action as quickly as possible in response to those cues [8,13,15].

Appropriate child feeding practices and behaviors of parents have a positive effect on growth of infants and young children [16]. For instance, an analysis of data sets from several Latin American countries demonstrated that appropriate breastfeeding and complementary feeding practices were positively associated with child height-for-age in most of the countries studied [17]. However, the majority of the literature on child feeding and parental practices are based on women in affluent societies and their emphases have been on child overweight or obesity [18-20]. Control over feeding appears to vary among cultures, socioeconomic status and child's gender [21]. Hence, results of the effect of different parental or caregiver feeding behaviors among various socio-cultural settings should be used cautiously.

Most studies carried out in Ethiopia have focused mainly on identifying factors associated with undernutrition instead of overweight or obesity [5,22]. To the best of our knowledge, no studies have been conducted to assess child feeding behavior of caregivers and the association between these behaviors and child growth and development, dietary intake, eating behavior or morbidity in Ethiopia. Current interventions mainly address the issue of what to feed young children instead of how to feed them [8]. The aim of this study was to identify the different types of child feeding behaviors (styles) of caregivers and the factors that influence these behaviors.

## Methods

### Study area and setting

A community based cross-sectional study was conducted in January 2009 in Derashe special district, Southern Ethiopia. Based on the 2007 census, the projected total population of the district in 2009 was 149,901 and the total household count was 29,980. Children 6-23 months of age constituted 6.7% of the total population. The livelihood of more than 92% of the district population is based on farming. The main crops grown in the area are maize, sorghum and teff (a species of *Eragrostis *native to Ethiopia).

### Sampling

The source population of this study included all residents of Derashe district who were caregivers of children aged 6-23 months and resided in the district for more than 6 months. The sample size was calculated by using the single population proportion formula. Assumptions for the calculation were that 50% of caregivers practiced a "*responsive*" feeding style (P) with a 95% confidence level and 5% tolerable error. The calculated sample size was 384. As a multistage sampling technique was employed to identify study subjects, a design effect of 2 was used to reach a sample size of 768 to which 10% was added for non-responses. Thus, the final sample size was 845.

A multistage stratified sampling technique was used to identify study subjects after the Kebeles (smallest administrative units) were stratified into urban and rural Kebeles. Based on the proportion of population by urban-rural residence [23], seven rural and one urban Kebele were randomly selected in the district. A census was conducted in the selected Kebeles to identify households with children aged 6-23 months and to generate a sampling frame. A total of 3021 children in this age group who were started with complementary food were identified and listed. The calculated sample (845) was proportionally allocated to the selected Kebeles based on the total number of caregivers of 6-23 months children in each Kebele and study subjects were identified by simple random sampling technique.

### Method of data collection

Data was collected by a face-to-face interview technique using the adapted Caregiver's Feeding Styles Questionnaire [24,25]. The questionnaire was first prepared in English and later translated into Amharic (the local language) and back translated into English to check for its conceptual equivalence. Prior to the actual data collection, the questionnaire was pre-tested using 5% of the sample from a similar population outside the study area. Twelve high school complete data collectors, with previous experience in nutritional surveys, were recruited and trained by the principal investigators on the study instrument and data collection procedures. Reliability of the measurements was assured by training two health officers from the district for close follow-up. The principal investigators constantly checked strict adherence to the standards agreed upon by all field workers.

### Measurement of caregivers' feeding styles

Data collectors asked the caregivers to respond to behavioral and belief items pertaining to each feeding style. However, many caregivers displayed behaviors and beliefs of more than one feeding style which made strict categorization into the three styles (controlling, laissez- faire or responsive) according to Birch and Fisher [11] difficult. Hence, those caregivers practicing a particular style most of the time for behavioral items, or agreeing or highly agreeing with a belief item, were considered as practicing responsive, controlling or laissez-faire feeding styles depending on the response to each behavioral and belief item. The behavioral and belief items of the questionnaire were adapted to the local context from similar studies conducted using a validated instrument.

### Data analysis

The data was analyzed using Stata version 11 (StataCorp, College Station, TX, USA). Nineteen caregivers were not found despite repeated visits. Sixty two questionnaires with inadequate information to measure caregiver feeding style were excluded making the total observation with complete information 764. Bivariate and multivariate multinomial logistic regression was employed to assess statistical associations between caregivers' feeding style and caregiver and child characteristics. Residence, age of caregiver and the study child, relation of the study child with the caregiver, educational status of the caregiver and the father of the study child, parity of the biological mother, birth interval between the study child and older sibling, frequency of breastfeeding and advice on child feeding by a health professional were entered as independent variables in the model. Responsive feeding style was used as a reference category in the model.

Variables which showed a significant association with caregivers' feeding style in the bivariate analysis, were entered into the multivariate multinomial logistic regression. Relative Risk Ratios (RRR), the relative risk ratios for the multinomial logistic model, were determined to assess whether the risk of the outcome falling in the comparison group (either controlling or laissez- faire feeding style) compared to the risk of the outcome falling in the reference group (responsive feeding style) increases or decreases with the independent variable. The p-value (P > |z|), is the probability that the z test statistic would be as extreme as, or more so, than what has been observed under the null hypothesis. If P > |z| is less than 0.05, the null hypothesis can be rejected and the RRR is considered significant at the 5% level.

### Ethical considerations

Ethical approval was obtained from the Ethical Review Committee of Jimma University. Informed consent was secured from all caregivers of study children before commencing the interview.

## Results

### General characteristics of caregivers and study children

Of the 845 sampled caregivers, 826 were successfully included in the study making the response rate 97.7%. Biological mothers accounted for 666 (80.6%) of caregivers, while 160 (19.4%) were other caregivers such as grandmothers, sisters and other relatives. Most of the caregivers (87.6%) were rural residents. Fifty percent of the respondents were 25-34 years of age. Seventy percent of the caregivers did not attend any formal education and 172 (71.7%) of those who attended school attended only elementary school. Three hundred fifty-nine (54.5%) of the biological mothers, who were at the same time caregivers, had given birth to only one child. Five hundred and sixty five (79.9%) received advice from health professionals about child feeding (Table 1).

**Table 1:** Characteristics of caregivers of study children in Derashe special district, Southern Ethiopia

Variables	Frequency	Percent
**Residence of the caregiver (n = 826)**		

Urban	103	12.5

Rural	723	87.5

**Age of the caregiver (years) (n = 826)**		

< 20	87	10.5

20-24	145	17.6

25-29	260	31.5

30-34	156	18.9

> = 35	178	21.5

**Relation of the study child with the caregiver (n = 826)**		

Mother	666	80.6

Others*	160	19.4

**Educational status of the caregiver (n = 826)**		

No formal education	582	70.5

Elementary	175	21.2

Above elementary	69	8.4

**Educational status of the father of study child (n = 826)**		

No formal education	376	45.5

Elementary	251	30.4

Above elementary	199	24.1

**Parity of the biological mother (n = 666)**		

Primiparous	118	17.7

Multiparous	341	51.2

Grand multiparous	207	31.1

**Advice on young child feeding by a health professional (n = 716)**		

Advised	565	78.9

Not advised	151	21.1

*Grandmothers, sisters, other relatives

The majority of interviewed caregivers (61.6%) had 12-23 month old children (Table 2). Biological mothers were the main caregivers of most of the 6-11 month old children (87%), while grandmothers were caregivers of 20 (6.4%) of these children. Mothers and grandmothers were the main caregivers of 396 (76.5%) and 64 (12.5%) of the 12-23 month old children, respectively (data not shown).

**Table 2:** Characteristics of the study children in Derashe special district, Southern Ethiopia

Variables	Frequency	Percent
**Age of the study child (n = 826)**		

6-8 months	188	22.8

9-11 months	129	15.6

12-23 months	509	61.6

**Sex (n = 826)**		

Male	417.0	50.5

Female	409.0	49.5

**Birth interval between the study and older sibling (n = 637)**		

Short birth interval	422	66.2

Optimal birth interval	215	33.8

**Frequency breast feeding in the last 24 hours (n = 666)**		

Less than recommended	432	64.9

Recommended	234	35.1

### Breastfeeding and complementary feeding practices of caregivers

Six hundred sixty three (80.2%) of the caregivers were breastfeeding at the time of the study. Among the caregivers who specified breastfeeding frequency, 64.9% were feeding their children less than eight times per day. Two hundred ninety three (38.7%) of the interviewed caregivers had started giving complementary foods or drinks other than breast milk (mostly water, gruel and other semisolid cereal-based preparations) to their infants before they reached 6 months of age. For half of the caregivers, the reason for introducing foods/drinks too early was the belief that breast milk alone is not sufficient at that age. The mean (± SD) complementary feeding frequency was 4.6 ± 1.3, 5.1 ± 1.4 and 6.2 ± 1.6 times per day for study children 6-8, 9-11 and 12-24 months of age, respectively. Thirty eight percent of the study children ate from a separate bowl (data not shown).

### Feeding styles of caregivers

The majority (75.7%) of caregivers fed their children mostly with a "responsive" feeding style, followed by "controlling" and "laissez-faire" in 98 (12.8%) and 88 (11.5%) of the caregivers, respectively. On the other hand, 660 (80.0%) and 670 (81.2%) of the caregivers replied that the study child refused to eat if he/she was not hungry and when he/she was ill, respectively. Seventy seven (9.3%), 52 (6.4%) and 7 (0.9%) of the caregivers replied that the study child refused to eat if he/she was forced to eat, if someone other than the caregiver was feeding him/her and if he/she was not encouraged to eat, respectively (data not shown).

In response to food refusal by the child, 512 (66.9%) of the caregivers encouraged their children to eat either verbally or physically. According to the responses of 405 (53.0%) of the caregivers, the children ate more when encouraged verbally, while 292 (38.2%) of the respondents replied that the children ate more when food was provided on a separate plate (Table 3). Five hundred eighty (70.3%) of the caregivers fed their sick child by verbal or physical encouragement. However, 182 (22.0%) of the caregivers force-fed their children during illness while 71 (8.7%) used other options to help their children eat under similar circumstances.

**Table 3:** Caregivers' feeding styles, responses to child's refusal to eat and conditions in which child eats more, Derashe special district, Southern Ethiopia

Variables	Frequency	Percent
**Caregivers' feeding style (n = 764)**		

Laissez-faire	88	11.5

Controlling	98	12.8

Responsive	578	75.7

Active responses for food refusal*		

Encourage him/her to eat verbally or physically	512	66.9

Offers the same food later	239	31.2

Offers alternative foods (favorite foods)	141	18.4

Forces the child to feed	84	11.1

Do not try any solutions	41	5.4

Condition in which the child eats more*		

When encouraged to eat	405	53.0

When food is presented in a separate plate	292	38.2

When the caregiver sits with the child while the child is eating	221	27.0

Forcing the child to eat	47	4.6

*There were multiple responses by a caregiver

### Predictors of caregiver feeding styles

#### Laissez-faire relative to responsive feeding style

The laissez-faire style is characterized by the lack of extra effort by the caregiver in making the child eat even if the child is at risk of undernutrition. Caregivers from rural villages were less likely (RRR = 0.16; 95% CI = 0.08-0.33) to practice a laissez-faire feeding style compared to a responsive feeding style given that the other variables in the model are kept constant. Caregivers other than biological mothers (RRR = 2.5; 95% CI = 1.43-4.38), who breastfed more than eight times during the previous day (RRR = 1.88; 95% CI = 1.03-3.41) and biological mothers with an optimal birth interval between study child and older sibling (RRR = 2.46; 95% CI = 1.38-4.40) were more likely to practice a laissez-faire feeding style compared to a responsive feeding style (Table 4).

**Table 4:** Multinomial logistic regression of predictors of feeding style of caregivers, Derashe special district, Southern Ethiopia

Caregivers' feeding style	RRR *	P > |z|	[95% Conf. Interval]	
**Laissez-faire**				

Rural residence by the caregiver	0.16	0.000	0.08	0.33

Caregiver other than a biological mother	2.50	0.001	1.43	4.38

Frequency of BF more than eight times in the previous 24 hours	1.88	0.038	1.03	3.41

Optimal birth interval between the study child and older sibling (36-60 months)	2.46	0.002	1.38	4.40

**Controlling**				

Caregiver other than a biological mother	1.86	0.02	1.10	3.15

Frequency of BF more than eight times in the previous 24 hours	1.70	0.043	1.02	2.85

Optimal birth interval between the study child and older sibling (36-60 months)	0.54	0.038	0.30	0.97

*RRR = Relative Risk Ratio; n = 764; The reference category is "Responsive feeding style"

#### Controlling relative to responsive feeding style

Caregivers with a controlling feeding style try to control the time, type and the amount of food the child eats. They may also practice force-feeding when the child refuses to eat. Caregivers who were not biological mothers, compared to biological mothers, were more likely (RRR = 1.86; 95% CI = 1.1-3.15) to practice a controlling feeding style compared to responsive feeding. Caregivers who breastfed more than eight times during the previous day compared to those feeding less frequently were more likely (RRR = 1.7; 95% CI = 1.02-2.85) to practice a controlling feeding style. Moreover, it was also found that biological mothers with an optimal birth interval between study child and older sibling were less likely (RRR = 0.55; 95% CI = 0.32-0.95) to practice a controlling feeding style compared to a responsive feeding style (Table 4).

## Discussion

This study examined three types of caregivers' feeding styles (laissez-fair, controlling, responsive) from a socio-demographic, child feeding and caregivers' behavior perspective. The majority of the studies investigating these three child feeding styles were carried out in developed countries and used a variety of techniques and instruments. This made direct comparisons of the findings from this study with other findings difficult.

In this study, 51% of the children were exclusively breastfed at 6 months of age. This is much higher than the findings reported in the Ethiopian Demographic and Health Survey 2005 (EDHS 2005). The EDHS 2005 report showed that only one in three children of 4-5 months of age were exclusively breastfed and that the rate of exclusive breastfeeding for those less than 6 months was 49% [5]. This might be because EDHS 2005 was a nationally representative survey with a wide range of exclusive breastfeeding proportions in different regions of Ethiopia.

The WHO recommends children of 6-8, 9-11 and 12-23 months of age to be fed 2-3, 3-4 and 3-4 times per day, respectively, with the addition of 1-2 snacks [9]. In this study, 95% of the children within each of these respective age groups received complementary foods as frequent or more than the WHO recommendation. The mean (± SD) complementary feeding frequency per day was 4.6 ± 2.0, 5.2 ± 1.8 and 5.2 ± 2.0 times for children in the respective age groups. This result was comparable to the findings from rural Malawi where the mean (± SD) complementary feeding frequency was 3.1 ± 1.2, 4.1 ± 1.1 and 4.4 ± 1.4 times, respectively [26]. The majority of caregivers were classified as practicing a responsive child feeding style (75.7%) which is consistent with the findings from a study in Vietnam [12].

In this study, 80.6% of caregivers were biological mothers. A study conducted in Nicaragua showed that mothers, as caregivers, were more likely than other caregivers to offer encouragement for eating [27]. Most of the caregivers in this study were rural dwellers but practiced a responsive feeding style most of the time. Two thirds of rural mothers in Bangladesh practiced active feeding ("behavior that encourages the child to eat or the mother to feed, either indirectly through words or directly through forcing food into the child's mouth") but only a third of them were responsive [28].

Caregivers' behavior in child feeding are part of the broader socio-cultural context of societies in different geographical locations [29]. Several studies suggested that appropriate child feeding practices have an impact on child nutritional status [10,15-17,20,30]. Responsive or active feeding was found to be more beneficial to the child in some low- or middle-income countries [15,27,31,32] and non-responsive feeding was associated with child BMI and overweight or obesity in high-income countries [20] though, cross-study definitions of responsive feeding were inconsistent. Responsiveness was also observed in reaction to a child's decreased appetite during diarrheal episodes in Guatemala [33] and Peru [34]; and low interest in food in Nicaragua [27]. However, recent reviews concluded that the effect of responsive feeding on child weight or growth was mixed and failed to isolate the effect of responsive feeding, as most of these studies were done together with broader health or nutrition education and food supplementation programs [15] and used cross-sectional designs [20]. Moreover, the mechanism of linking feeding behavior and child weight or growth differs between low- and middle-income and high-income countries [15,20].

In developing countries, there is very little information on the practice of controlling or laissez-faire feeding and implications for child nutrition. In Ghana, it was found that using force during feeding was common among caregivers with negative deviance in child feeding and had a direct effect on child's nutrient intake [32]. In Nigeria, force-feeding and pressurizing was followed by food refusal by the child [35]. In Vietnam, non-responsive feeding, characterized by pressure or force-feeding, was associated with the child's rejection of the food [36]. A recent study in Malawi revealed that controlling feeding behavior was negatively associated with the acceptance of food by the child [37].

Unlike the present study, a laissez-faire feeding style was the most frequently observed behavior among caregivers in Peru and Guatemala [33,34]. Encouraging feeding in these circumstances was a response only to a decrease in child food intake because of illness and hence was not pro-active.

This study did not assess the outcome of a particular feeding style practiced by caregivers, though the available literature on feeding styles underlined the association of feeding style with child growth and development, acceptance of food or child eating behavior and overweight or obesity [14,30,38-41]. As a limitation of the cross-sectional study design, temporality could not be established. Hence, it is difficult to determine whether a positive child response to the provided food leads to responsive feeding by caregivers or vice versa [15]. Nevertheless, behavior at any point in time could reflect prior caregiver-child interactions. The results of this study are also dependent on the reporting of the caregiver. Most studies used additional observations through videotaping of caregivers feeding behaviors which can increase the validity of the finding from interview-based studies [38].

## Conclusions and Recommendations

Responsive feeding was the commonest style practiced by the studied caregivers. In this study, rural residence and being a biological mother were found to be independent predictors of responsive feeding after controlling for potential confounders. As this study was the first of its kind in the country, more research is needed to check the validity and reliability of the Caregiver's Feeding Styles Questionnaire in Ethiopia and to evaluate the effect of caregivers' feeding style on child dietary intake and nutritional status in different socio-cultural settings within the country. Moreover, longitudinal research starting from early infancy is recommended to see the factors affecting caregiver's child feeding behavior and other care practices.

## Competing interests

The authors declare that they have no competing interests.

## Authors' contributions

**MW, TA **and **MW **designed the study, analyzed the data, drafted the manuscript, and hence had equal contribution. All authors read and approved the final manuscript.

## Pre-publication history

The pre-publication history for this paper can be accessed here:

http://www.biomedcentral.com/1471-2458/12/235/prepub
